# *Klebsiella pneumoniae* in Gastrointestinal Tract and Pyogenic Liver Abscess

**DOI:** 10.3201/eid1808.111053

**Published:** 2012-08

**Authors:** Chang-Phone Fung, Yi-Tsung Lin, Jung-Chung Lin, Te-Li Chen, Kuo-Ming Yeh, Feng-Yee Chang, Han-Chuan Chuang, Hau-Shin Wu, Chih-Peng Tseng, L. Kristopher Siu

**Affiliations:** Taipei Veterans General Hospital, Taipei, Taiwan (C.P. Fung, Y.T. Lin, T.L. Chen, H.C. Chuang, H.S. Wu, C.P. Tseng);; National Yang-Ming University, Taipei (C.P. Fung, Y.T. Lin, T.L. Chen, H.C. Chuang, H.S. Wu, C.P. Tseng);; National Defense Medical Center, Taipei (J.C. Lin, K.M. Yeh, F. Y. Chang);; National Health Research Institutes, Zhunan, Taiwan (L.K. Siu);; and China Medical University, Taichung, Taiwan (L.K. Siu)

**Keywords:** Klebsiella pneumoniae, capsular serotypes, gastrointestinal tract, pyogenic liver abscess, endogenous infection, bacteria, virulence, pulsed-field gel electrophoresis, Taiwan

## Abstract

To determine the role of gastrointestinal carriage in *Klebsiella pneumoniae* liver abscess, we studied 43 patients. Bacterial isolates from liver and fecal samples from 10 patients with this condition and 7 healthy carriers showed identical serotypes and genotypes with the same virulence. This finding indicated that gastrointestinal carriage is a predisposing factor for liver abscess.

*Klebsiella pneumoniae* has emerged as the predominant cause of pyogenic liver abscess in Taiwan and other countries in eastern and Southeast Asia (*1**–**4*). This condition is frequently associated with severe complications, including septic endophthalmitis and other extrahepatic lesions, especially in patients with diabetes (*1*).

In Taiwan, the annual incidence of pyogenic liver abscess has increased steadily from 11.15 cases/100,000 persons in 1996 to 17.59 cases/100,000 persons in 2004, and *K. pneumoniae* was found in 79.9% of culture-positive cases (*5*). We showed that >3,000 new cases of pyogenic liver abscess were found each year during 2005–2008 (*6*). However, the pathogenesis of *K. pneumoniae* liver abscess remains unclear, and the source of endogenous or exogenous infections has been debated.

To determine whether strains recovered from liver aspirate samples originated from gastrointestinal flora of patients, we investigated isolates from liver aspirate, nasal swab, saliva, and fecal samples by using genomic analysis. Using serotyping and molecular typing methods, we systematically investigated the association between isolates from liver aspirates and those from other body sites in patients with *K. pneumoniae* liver abscess. We also investigated *K. pneumoniae* isolates from healthy carriers that were genetically similar to liver abscess isolates to assess whether colonization of virulent *K. pneumoniae* occurs in these persons, which could subsequently lead to development of liver abscess.

## The Study

During January 2009–December 2010, a total of 43 adult patients (mean age 68.2 years) with liver aspirate cultures positive for *K. pneumoniae* in Taipei Veterans General Hospital were consecutively enrolled in the study. All cases of *K. pneumoniae* liver abscess were community acquired. Clinical characteristics of patients are shown in Table 1.

**Table 1:** Clinical characteristics of 43 patients with *Klebsiella pneumoniae* liver abscess, Taiwan, January 2009–December 2010*

Characteristic	No. (%) patients		p value
	Serotype K1/K2, n = 33	Non–K1/K2,†(n = 10	
Sex			0.89
M	19 (57.6)	6 (60.0)	
F	14 (42.4)	4 (40.0)	
Symptom/sign			
Fever	33 (100.0)	10 (100.0)	
Chills	20 (60.6)	4 (40.0)	0.25
RUQ pain/tenderness in abdomen	31 (94.0)	9 (90.0)	0.67
Nausea/vomiting	10 (30.3)	3 (30.0)	0.99
Leukocytosis, >10^3^ cells/μL	32 (97.0)	10 (100.0)	1.00
Complication with distal metastasis	3 (9.1)	0	0.572
Endophthalmitis	1 (3.0)	0	
Meningitis	1 (3.0)	0	
Lung abscess	1 (3.0)	0	
Underlying disease			
Diabetes mellitus	20 (60.7)	5 (50.0)	0.55
Alcoholism	2 (6.0)	1 (10.0)	0.59
Malignancy	4 (12.1)	3 (30.0)	0.13
CVA	2 (6.0)	1(10.0)	0.59
Biliary tract diseases	8 (24.2)	5 (50.0)	0.12
Liver cirrhosis	2 (6.0)	0	1.00
COPD	1 (3.0)	0	1.00
Chronic renal insufficiency	7 (21.2)	2 (20.0)	0.93
HBsAg positive	2 (6.0)	0	1.00
Deaths	4 (12.1)	0	0.593

*Mean ages of patients were 67.3 for those infected with serotype K1/K2 and 68.6 y for those infected with non-K1/K2. RUQ, right upper quadrant; CVA, cerebrovascular accident; COPD, chronic obstructive pulmonary disease; HBsAg, hepatitis B surface antigen. †Patients with serotypes K15 (1), K19 (3), K31 (1), K46 (1), K54 (2) and nontypeable (2).

To determine whether *K. pneumoniae* liver abscess originated from the gastrointestinal tract of patients, we concomitantly tested all liver aspirate, saliva, nasal swab, fecal, and blood samples by using bacterial culture before patients were treated with antimicrobial drugs. A total of 125 *K. pneumoniae* isolates from 43 patients were obtained. Information on culture-positive sites is shown in the Technical Appendix Table. To compare virulence and genetic relatedness of *K. pneumoniae* from liver abscess patients and healthy carriers, we obtained 1,000 *K. pneumoniae* isolates from fecal samples of asymptomatic adults during routine physical examinations at the Tri-Service General Hospital (Taipei, Taiwan).

All clinical *K. pneumoniae* isolates were serotyped by using countercurrent immunoelectrophoresis with serotype K antiserum. Isolates with serotypes K1 and K2 were confirmed by PCR (*7*). All K1 isolates were screened for CC23 representatives by detection of the *allS* gene by using PCR as described (*7*). Seroepidemiologic study of *K. pneumoniae* isolates from liver abscess patients showed that serotypes K1 and K2 were predominant, accounting for 61% (26/43) and 16% (7/43) of all isolates, respectively.

All K1 isolates had the *allS* gene, which is consistent with results of a study that showed that the virulent K1 clone of CC23 is associated pyogenic liver abscess (*7*). Although there was no difference in clinical characteristics between K1/K2 and non-K1/K2 patients, complications of distal septic metastasis (9%) and death (mortality rate 9%) were found for the K1/K2 group (Table 1).

**Figure 1:**
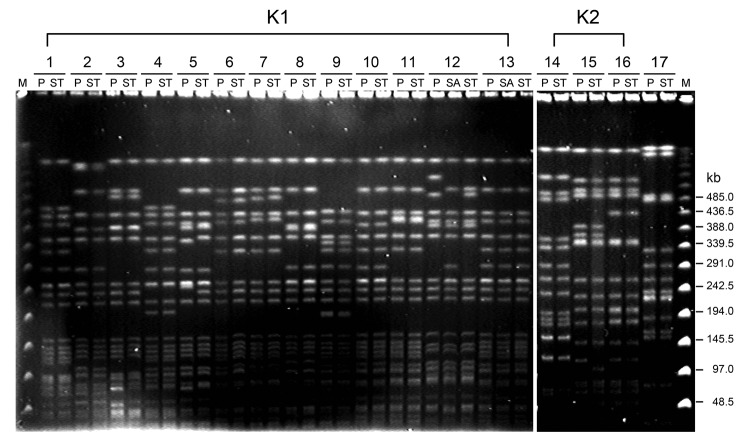
Pulsed-field gel electrophoresis of randomly selected isolates of *Klebsiella pneumoniae* from 17 patients with liver abscess, Taiwan, January 2009–December 2010. DNA fragments were subjected to electrophoresis after digestion with *Xba*I. Lanes 1–13, serotype K1 isolates; lanes 14–16, serotype K2 isolates; lane 17, serotype non-K1/K2 isolates. M, molecular mass marker; P, liver aspirate; ST, stool; SA, saliva.

A total of 17 randomly selected pairs of representative *K. pneumoniae* isolates (from liver aspirate, saliva, and fecal samples), including serotypes K1 (13 isolates), K2 (3 isolates), and non-K1/K2 (1 isolate), from patients with liver abscess were subjected to pulsed-field gel electrophoresis (PFGE) analysis with *Xba*I. All liver aspirate isolates had a PFGE profile identical or closely related to those of fecal or saliva samples from the same patient (Figure 1). Isolates from different patients that belonged to serotypes K1 and K2 were distinguishable from each another, indicating epidemiologic unrelatedness of these strains.

**Figure 2:**
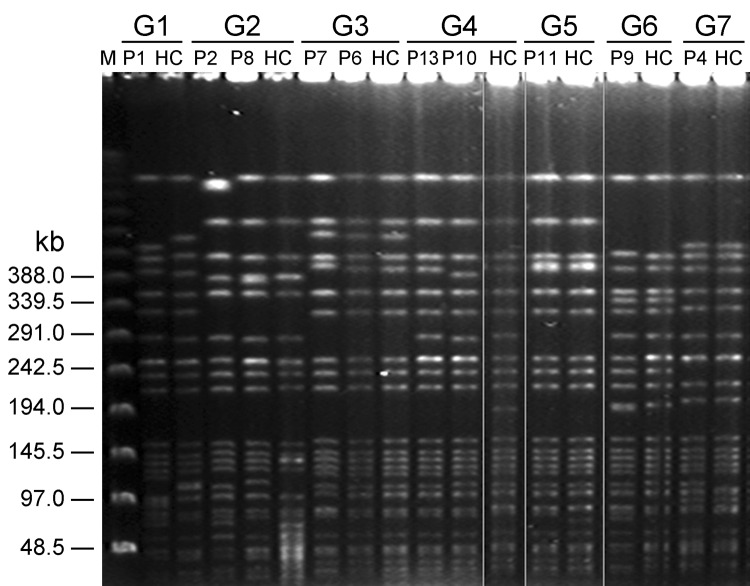
Pulsed-field gel electrophoresis of *Klebsiella pneumoniae* isolates from fecal samples of 7 patient groups with liver abscess and healthy carriers, Taiwan, January 2009–December 2010. G, patient group; M, molecular mass marker; P, patient; HC, healthy carrier.

Among 17 patients, PFGE matching of liver aspirate *K. pneumoniae* isolates to isolates from fecal samples of 1,000 healthy adults was performed by using computer program analysis. PFGE showed that 7 groups of serotype K1 *K. pneumoniae* isolated from fecal samples from 10 patients and 7 healthy carriers had identical and >90% similarity in PFGE patterns (Figure 2). No serotype K2 or non-K1/K2 isolates could be matched with other isolates from healthy carriers.

The *rmpA* and aerobactin virulence genes were detected in all K1/K2 isolates (*8*). In vitro and in vivo assays to assess virulence were performed by using neutrophil phagocytosis and serum resistance assays as described (*9*). PCR showed that *rmpA* and aerobactin virulence genes were present in all 17 matched isolates from liver aspirate specimens and healthy carriers. Virulence assessment demonstrated that groups 1–6 (except patient 7) were resistant to phagocytosis and showed evidence of serum resistance (Table 2). In mouse lethality assays, various 50% lethal dose (LD_50_) were observed among 7 groups of isolates from liver aspirates. Similar LD_50_ values were observed among all 7 groups of isolates, indicating no difference in virulence between isolates from patients and those from healthy carriers, and that the healthy adults carried the virulent strains in their intestines

**Table 2:** Virulence of serotype K1 *Klebsiella pneumoniae* isolates and clonal relationship from patients with liver abscess and healthy carriers, January 2009–December 2010, Taiwan*

Group no.†	Source	Serum resistance	Phagocytosis, %‡	Mice LD_50_, CFU
1	P1	R	30.9	<2.6 × 10^2^
	HC-1	R	31.0	2.3 × 10^3^
2	P2	R	24.2	<2.9 × 10^2^
	P8	R	18.3	<1.5 × 10^2^
	HC-2	R	8.8	3.5 × 10^2^
3	P7	S	86.9	>1.7 × 10^5^
	P6	R	29.7	2.2 × 10^2^
	HC-3	R	35.6	3.0 × 10^2^
4	P13	R	37.6	3.5 × 10^2^
	P10	R	30.7	<3.8 × 10^2^
	HC-4	R	18.8	<3.0 × 10^2^
5	P11	R	20.4	2.3 × 10^3^
	HC-5	R	13.5	<2.4 × 10^2^
6	P9	R	17.8	3.4 × 10^3^
	HC-6	R	31.7	1.7 × 10^3^
7	P4	S	35.6	1.9 × 10^4^
	HC-7	S	33.1	5.2 × 10^3^

*All groups were positive for the *rmpA* and aerobactin genes. LD_50_, 50% lethal dose; P, patient; R, resistant: HC, health carrier; S, sensitive. †Isolates with an identical or clonally related pulsed-field gel electrophoresis profile from liver aspirate and feces of healthy carriers. ‡Percentage of *K. pneumoniae* phagocytosed by neutrophils after 15 min.

## Conclusions

Molecular typing of the *K. pneumoniae* strains from different patients showed different patterns, indicating epidemiologic unrelatedness of these strains, a finding that excludes a common origin of *K. pneumoniae* and transmission between patients. All PFGE profiles for liver aspirate isolates were identical or clonally related to those for isolates from fecal samples, suggesting that these infections originated from patient flora.

A previous study showed that serotype K1 and K2 isolates with aerobactin and *rampA* genes were more virulent (*10*). We found 3 isolates from patient 7, patient 4, and healthy carrier 7 that had aerobactin and *rmpA* genes and were less virulent than other matched groups. This finding is compatible with results of our previous investigation (*8*). Thus, development of *K. pneumoniae* liver abscesses probably results from a combination of virulence determinants rather than a single factor (*8*). Furthermore, we found that 6 groups of serotype K1 *K. pneumoniae* strains isolated from liver abscess patients (not patient 7) and 6 from healthy carriers had identical PFGE profiles with the same virulence-associated genes and similar LD_50_ values. This finding indicated that healthy adults had virulent strains in their gastrointestinal tracts.

Although *K. pneumoniae* liver abscess might be induced by inhalation of *K. pneumoniae* and a bacteremic phase, we showed that none of 49 patients with community-acquired pneumonia and bacteremia caused by *K. pneumoniae* showed development of *K. pneumoniae* liver abscess concomitantly (*11*). A study from Japan reported familial spread of a virulent K1 clone causing *K. pneumoniae* liver abscess (*12*). In this study, 1 family member without *K. pneumoniae* liver abscess had the same clone in her feces (*12*). Thus, the route of entry is probably from the gastrointestinal tract. An animal study demonstrated that *K. pneumoniae* strains with genetic regulatory networks for translocation have the ability to cross the intestinal barrier and cause liver abscess (*13*).

Carrier rates of *K. pneumoniae* have differed considerably among studies. The rate of detection in fecal samples from healthy persons ranged from 19.4% to 38% in studies in Europe (*14*). In a recent investigation, we reported a fecal carriage rate of *K. pneumoniae* in healthy adults of 75% and high prevalence (23%, 17/76) of serotype K1/K2 isolates among typeable strains in Taiwan (*15*). The high carriage of virulent *K. pneumoniae* strains in feces is probably the reason why there are so many cases of *K. pneumoniae* liver abscess in Taiwan.

Our study demonstrated that intestinal colonization of virulent type *K. pneumoniae* is highly associated with pyogenic liver abscess. Early detection of colonization by *K. pneumoniae*, especially serotypes K1 and K2 in patients with diabetes, might help with making treatment decisions for infected patients.

Technical Appendix Culture-positive sites for *lebsiella pneumoniae* from 43 liver abscess patients, Taiwan, January 2009–December 2010.
