# Supplementary material for: Klebsiella pneumoniae in Gastrointestinal Tract and Pyogenic Liver Abscess
Source: Emerg Infect Dis. 2012 Aug;18(8):1322–5. doi: 10.3201/eid1808.111053 (PMC3414011; doi:10.3201/eid1808.111053)
Supplement: Technical Appendix — Culture-positive sites for lebsiella pneumoniae from 43 liver abscess patients, Taiwan, January 2009–December 2010. [file 11-1053_Techapp-s1.pdf]

Article DOI: http://dx.doi.org/10.3201/eid1808.111053

Klebsiella pneumoniae in Gastrointestinal Tract and Pyogenic Liver Abscess

Technical Appendix

Technical Appendix Table. Culture-positive sites for *Klebsiella pneumoniae* from 43 liver abscess patients, Taiwan, January 2009–December 2010*

Patient	Liver	Feces	Blood	Nasal swab	Saliva	Other†	Serotype
1	+	+	+	–	+	–	K1
2	+	+	+	–	–	–	K1
3	+	+	+	+	–	–	K1
4	+	+	+	–	–	+	K1
5	+	+	+	–	+	–	K1
6	+	+	+	–	–	–	K1
7	+	+	+	–	–	–	K1
8	+	+	–	+	–	–	K1
9	+	+	+	–	–	+	K1
10	+	–	+	–	+	–	K1
11	+	+	+	–	–	–	K1
12	+	+	+	–	+	–	K1
13	+	+	–	–	–	–	K1
14	+	+	+	–	–	–	K1
15	+	+	+	–	+	–	K1
16	+	+	+	–	–	–	K1
17	+	+	+	–	–	–	K1
18	+	+	–	–	–	–	K1
19	+	+	+	–	–	–	K1
20	+	+	–	–	–	–	K1
21	+	+	+	–	–	–	K1
22	+	+	–	–	–	–	K1
23	+	+	+	+	–	+	K1

Patient	Liver	Feces	Blood	Nasal swab	Saliva	Other†	Serotype
24	+	+	+	–	–	–	K1
25	+	+	–	–	–	–	K1
26	+	+	+	–	–	–	K1
27	+	–	+	–	–	–	K2
28	+	+	–	+	–	–	K2
29	+	+	+	–	–	–	K2
30	+	–	+	–	+	–	K2
31	+	+	+	–	+	–	K2
32	+	+	+	–	–	–	K2
33	+	+	+	+	–	–	K2
34	+	+	–	–	–	–	K5
35	+	+	+	–	–	–	K19
36	+	+	+	–	+	–	K19
37	+	+	+	–	–	–	K19
38	+	–	–	–	–	–	K31
39	+	+	+	–	–	–	K46
40	+	+	–	–	–	–	K54
41	+	–	–	–	+	–	K54
42	+	+	–	–	–	–	NT
43	+	–	+	–	–	–	NT
Total	43	35	30	5	9	3	NA

*+, positive; –, negative;. NT, nontypeable; NA, not applicable.

†Eye aspirate from patient 4, cerebrospinal fluid from patient 9, and lung abscess from patient 23.
